# Inward migration of a dedicated hepaticogastrostomy plastic stent into the bile duct after hepaticojejunostomy

**DOI:** 10.1055/a-2845-0721

**Published:** 2026-04-20

**Authors:** Akito Furuta, Keijiro Ueda, Kazuhide Matsumoto, Masatoshi Murakami, Nao Fujimori, Yoshihiro Ogawa

**Affiliations:** 1 145181 Department of Medicine and Bioregulatory Science, Kyushu University Hospital, Fukuoka, Japan

**Fig. 1:**
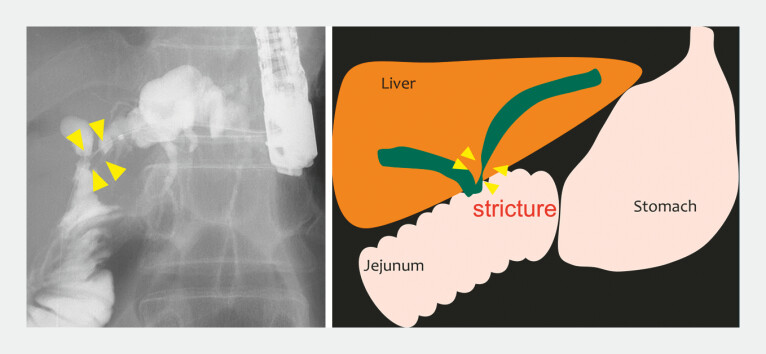
An anastomotic stricture was identified between the left hepatic duct and the jejunum.

**Fig. 2:**
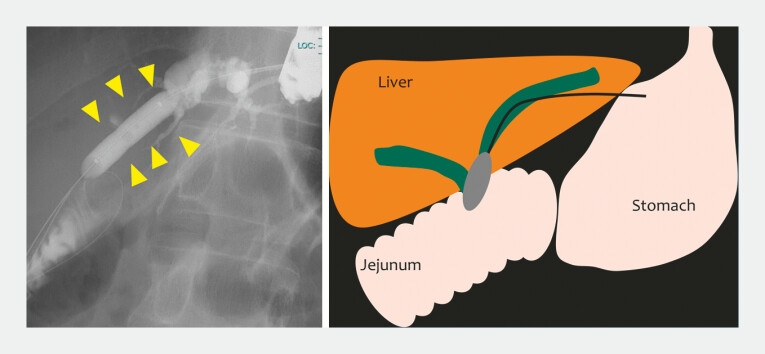
In the third session, the notch of the balloon was no longer visible, indicating adequate dilation.

The usefulness of a dedicated hepaticogastrostomy (HGS) plastic stent (TYPE-IT stent; Gadelius Medical, Tokyo, Japan) for endoscopic ultrasound-guided hepaticogastrostomy (EUS-HGS) has been reported 1 2 . However, to date, no reports have been published on the inward migration of dedicated HGS plastic stents. Here, we report a rare case of post-hepaticojejunostomy, in which a dedicated HGS plastic stent placed from the stomach through the bile duct and into the jejunum had migrated entirely into the bile duct ( Video 1 ). A 43-year-old man underwent extrahepatic bile duct resection and hepaticojejunostomy for congenital biliary dilatation. Nine years later, he developed cholangitis and underwent EUS-HGS with the placement of a dedicated HGS plastic stent. An anastomotic stricture was identified between the left hepatic duct and the jejunum ( Fig. 1 ). Balloon dilation using an 8-mm balloon was performed every 3 months. In the third session, the notch of the balloon was no longer visible, indicating adequate dilation ( Fig. 2 ), and two dedicated HGS plastic stents were placed from the stomach to the jejunum across the dilated anastomotic stricture.

Reintervention for the inward migration of a dedicated HGS plastic stent into the bile duct in a patient with post-hepaticojejunostomy. HGS, hepaticogastrostomy.Video 1

**Fig. 3:**
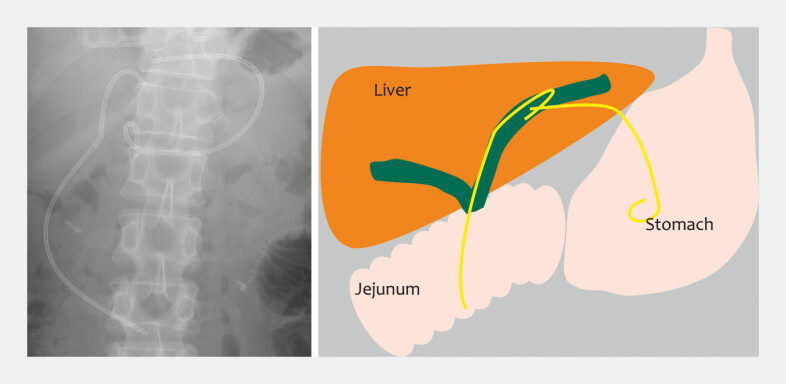
Abdominal radiography revealed that one of the stents had migrated into the biliary system.

**Fig. 4:**
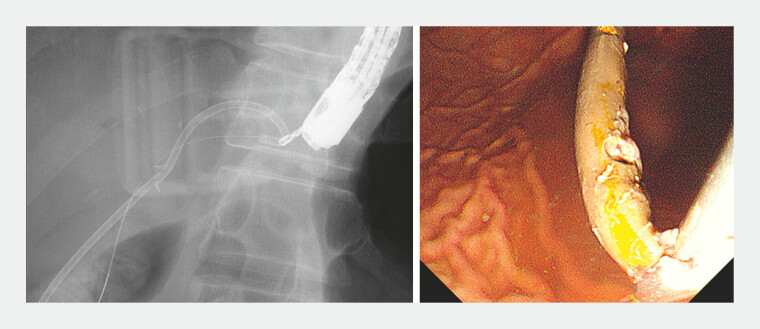
The migrated stent was successfully removed using the rotatable grasping forceps.

Approximately 3 months later, the patient presented with abdominal pain. Abdominal radiography revealed that one of the stents had migrated into the biliary system ( Fig. 3 ). An emergency endoscopic procedure was performed, during which a guidewire was advanced through the EUS-HGS tract, and the migrated stent was successfully removed using rotatable grasping forceps ( Fig. 4 ). Two double-pigtail plastic stents were placed, and the procedure was completed without complications. The patient recovered without further symptoms, and no additional episodes of stent migration occurred during the follow-up period. This case emphasizes that endoscopists should be cautious about the risk of inward stent migration, despite the use of dedicated HGS plastic stents specifically designed to prevent such complications.

Endoscopy_UCTN_Code_CPL_1AL_2AD
